# 3-[(3-Benzoyl-4-hy­droxy-1,1-dioxo-2*H*-1λ^6^,2-benzothia­zin-2-yl)meth­yl]benzo­nitrile

**DOI:** 10.1107/S1600536811052706

**Published:** 2011-12-10

**Authors:** Nazia Sattar, Hamid Latif Siddiqui, Tanvir Hussain, Sana Aslam, Masood Parvez

**Affiliations:** a Institute of Chemistry, University of the Punjab, Lahore 54590, Pakistan; b Department of Chemistry, The University of Calgary, 2500 University Drive NW, Calgary, Alberta, Canada T2N 1N4

## Abstract

There are two independent mol­ecules in the asymmetric unit of the title compound, C_23_H_17_N_2_O_4_S, with significant differences in their conformations, *e.g.* the benzene rings of the benzothia­zine and benzonitrile units are inclined at 28.19 (10) and 17.89 (7)° in the two mol­ecules, with the centroids of the rings separated by 3.975 (2) and 3.637 (2) Å, respectively. Moreover, the N—C—C—C torsion angles involving the benzoyl group are 14.3 (5) and 8.2 (5)° in the two mol­ecules, showing different degrees of rotation of this group. In both mol­ecules, the heterocyclic thia­zine rings adopt half-chair conformations, with the S and N atoms displaced by 0.427 (6) and 0.365 (6) Å, respectively, in one mol­ecule and by 0.356 (6) and 0.432 (6) Å, respectively, in the other, on opposite sides of the mean planes formed by the remaining ring atoms. The crystal structure is stabilized by inter­molecular C—H⋯O hydrogen bonds and further consolidated by intra­molecular O—H⋯O hydrogen bonds.

## Related literature

For the biological activity of benzothia­zine derivatives, see: Ahmad *et al.* (2010 ▶). For related structures, see: Siddiqui *et al.* (2008 ▶).
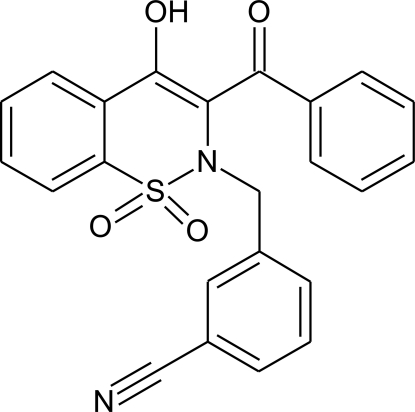


## Experimental

### 

#### Crystal data

C_23_H_16_N_2_O_4_S
*M* *_r_* = 416.44
Orthorhombic, 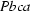

*a* = 12.2900 (2) Å
*b* = 24.7970 (6) Å
*c* = 25.6080 (6) Å
*V* = 7804.2 (3) Å^3^
*Z* = 16
Mo *K*α radiation
μ = 0.20 mm^−1^
*T* = 173 K
0.20 × 0.12 × 0.12 mm

#### Data collection

Nonius KappaCCD diffractometer
Absorption correction: multi-scan (*SORTAV*; Blessing, 1997 ▶) *T*_min_ = 0.961, *T*_max_ = 0.976
16724 measured reflections
8869 independent reflections
6212 reflections with *I* > 2σ(*I*)
*R*_int_ = 0.058

#### Refinement

*R*[*F* ^2^ > 2σ(*F* ^2^)] = 0.079
*wR*(*F* ^2^) = 0.164
*S* = 1.13
8869 reflections
543 parameters
H-atom parameters constrained
Δρ_max_ = 0.53 e Å^−3^
Δρ_min_ = −0.45 e Å^−3^

### 

Data collection: *COLLECT* (Hooft, 1998 ▶); cell refinement: *DENZO* (Otwinowski & Minor, 1997 ▶); data reduction: *SCALEPACK* (Otwinowski & Minor, 1997 ▶); program(s) used to solve structure: *SHELXS97* (Sheldrick, 2008 ▶); program(s) used to refine structure: *SHELXL97* (Sheldrick, 2008 ▶); molecular graphics: *Mercury* (Macrae *et al.*, 2008 ▶) and *ORTEP-3 for Windows* (Farrugia, 1997 ▶); software used to prepare material for publication: *SHELXL97*.

## Supplementary Material

Crystal structure: contains datablock(s) global, I. DOI: 10.1107/S1600536811052706/zl2435sup1.cif

Structure factors: contains datablock(s) I. DOI: 10.1107/S1600536811052706/zl2435Isup2.hkl

Supplementary material file. DOI: 10.1107/S1600536811052706/zl2435Isup3.cml

Additional supplementary materials:  crystallographic information; 3D view; checkCIF report

## Figures and Tables

**Table 1:** Hydrogen-bond geometry (Å, °)

*D*—H⋯*A*	*D*—H	H⋯*A*	*D*⋯*A*	*D*—H⋯*A*
C16—H16*A*⋯O3^i^	0.99	2.57	3.382 (4)	139
C16—H16*B*⋯O1^i^	0.99	2.60	3.456 (4)	145
C28—H28⋯O2^ii^	0.95	2.45	3.188 (4)	135
O3—H3*O*⋯O4	0.84	1.76	2.503 (4)	146
O7—H7*O*⋯O8	0.84	1.73	2.484 (4)	148

Symmetry codes: (i) 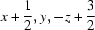; (ii) 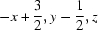.

**Fig. 1.**
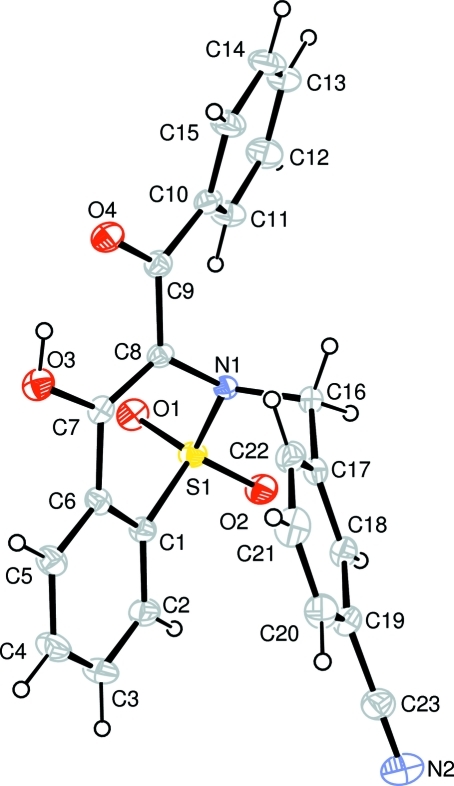
Molecule A of the title compound with displacement ellipsoids plotted at 30% probability level (Farrugia, 1997).

**Fig. 2.**
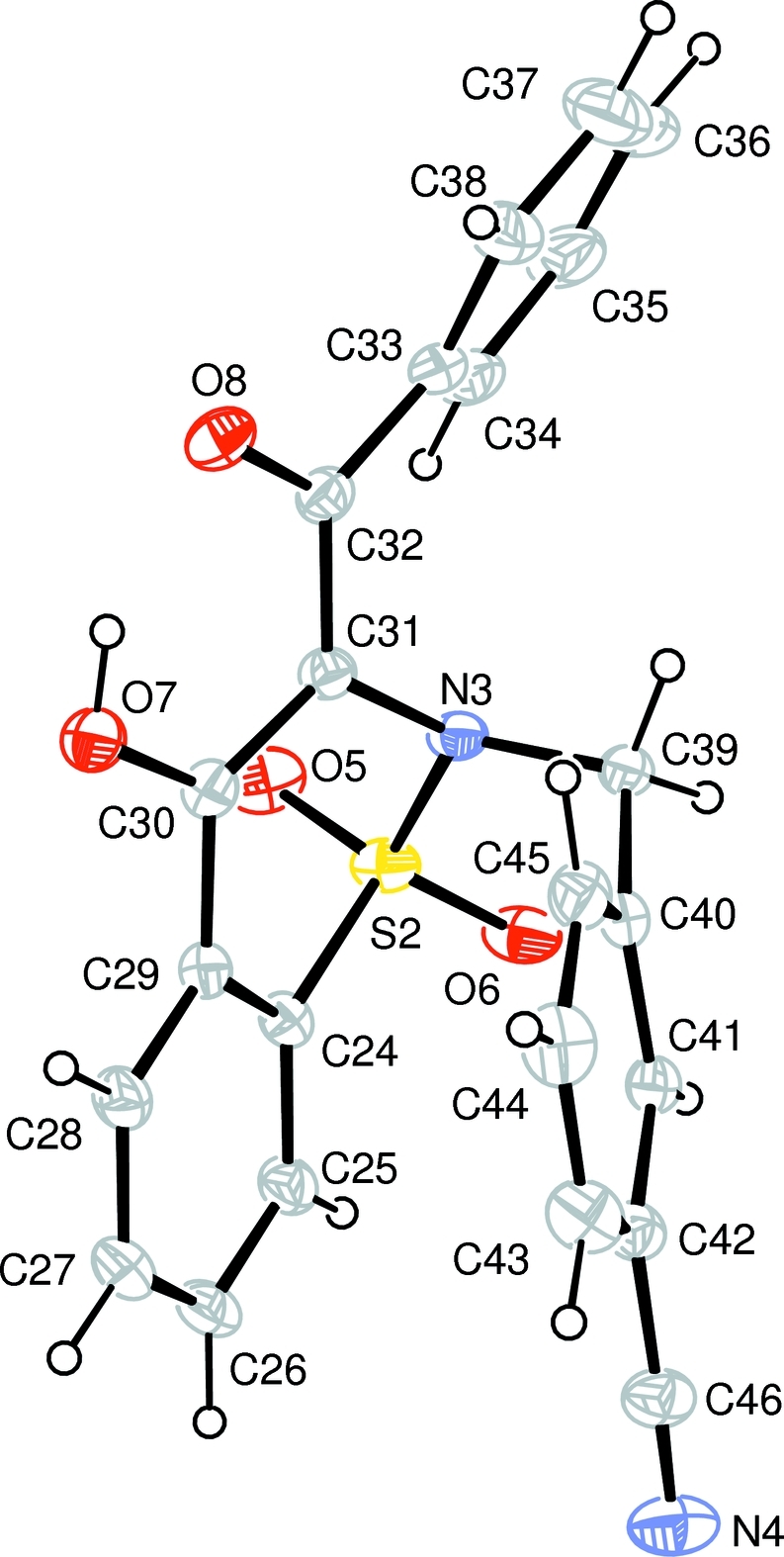
Molecule B of the title compound with displacement ellipsoids plotted at 30% probability level (Farrugia, 1997).

**Fig. 3.**
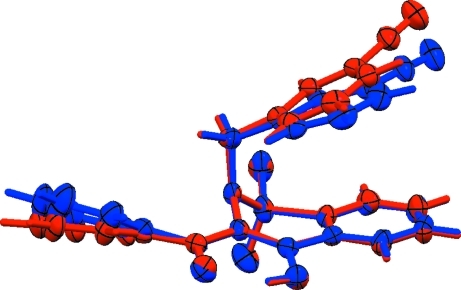
A plot of the molecule A (red) overlapping molecule B (blue) plotted with the aid of Mercury (Macrae et al., 2008) showing the conformational differences in the two molecules.

**Fig. 4.**
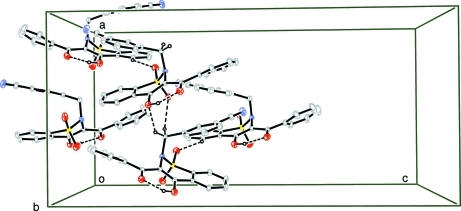
A partial unit cell packing diagram of the title compound showing hydrogen bonding interactions drwan as dashed lines. H-atoms not involved in H-bonding interactions have been excluded.

## supplementary crystallographic information

### Comment

In continuation of our research on the synthesis of biologically active benzothiazine derivatives (Ahmad *et al.*, 2010), we now report the synthesis and crystal structure of the title compound in this article.

There are two independent molecules in an asymmetric unit of the title compound, labeled as molecules A (Fig. 1) and B (Fig. 2) containing the S1 and S2 atoms, respectively. There are significant differences in the conformations of the two molecules, *e.g.*, the benzene rings of the benzothiazine and benzonitrile moieties are inclined at 28.19 (10) and 17.89 (7)° in the molecules A and B with centroids of the rings separated by 3.975 (2) and 3.637 (2) Å, respectively. Moreover, the torsion angles N1–C8–C9–C10 and N3–C31–C32–C33 in molecules A and B are 14.3 (5) and 8.2 (5)°, respectively, showing different degrees of rotation of these groups (Fig. 3). In both molecules, the heterocyclic thiazine rings adopt half chair conformations with atoms S and N displaced by 0.427 (6) and 0.365 (6) Å, in molecule A and 0.356 (6) and 0.432 (6) Å, in molecule B, respectively, on the opposite sides from the mean planes formed by the remaining ring atoms. The bond distances and angles in both molecules agree very well with the corresponding bond distances and angles reported in closely related compounds (Siddiqui *et al.*, 2008).

The methylene H-atoms bonded to C16 in molecule A are hydrogen bonded to O1 and O3 of two symmetry related molecules A (Tab. 1 and Fig. 4). On the other hand, an aryl H-atom, H28 of molecule B, is hydrogen bonded to O2 of molecule A. The structure is consolidated by intramolecular interactions of the types O—H···O, C—H···N and C—H···O.

### Experimental

An aqueous solution of sodium hydroxide (0.4 g, 9.96 mmol) was added to a solution of 3-benzoyl-4-hydroxy-2*H*-1,2-benzothiazine 1,1-dioxide (1.5 g, 4.9 mmol) in acetone (15 ml). 3-(Bromomethyl)benzonitrile (1.17 g, 5.98 mmol) was added with stirring and the reaction mixture was ultrasonicated for 15 minutes at 318 K. The completion of reaction was monitored with the help of thin layer chromatography (TLC). The contents of the flask were acidified to pH 3.0 by HCl (5%). Yellow precipitates of the title compound were filtered off and washed with excess of distilled water. Crystals suitable for crystallographic study were grown from methanol at room temperature. Yield = 1.93 g, 93.23%; m.p. = 444 - 446 K.

### Refinement

Though all the H atoms could be distinguished in the difference Fourier map the H-atoms were included at geometrically idealized positions and refined in riding-model approximation with the following constraints: O—H = 0.84, C—H = 0.95 and 0.99 Å, for aryl and methylene H-atoms, respectively. The *U*_iso_(H) were allowed at 1.2*U*_eq_(C/O). The final difference map was essentially featurless.

### Crystal data

**Table:**

C_23_H_16_N_2_O_4_S	*F*(000) = 3456
*M**_r_* = 416.44	*D*_x_ = 1.418 Mg m^−^^3^
Orthorhombic, *P**b**c**a*	Mo *K*α radiation, λ = 0.71073 Å
Hall symbol: -P 2ac 2ab	Cell parameters from 9408 reflections
*a* = 12.2900 (2) Å	θ = 1.0–27.5°
*b* = 24.7970 (6) Å	µ = 0.20 mm^−^^1^
*c* = 25.6080 (6) Å	*T* = 173 K
*V* = 7804.2 (3) Å^3^	Block, yellow
*Z* = 16	0.20 × 0.12 × 0.12 mm

### Data collection

**Table:**

Nonius KappaCCD diffractometer	8869 independent reflections
Radiation source: fine-focus sealed tube	6212 reflections with *I* > 2σ(*I*)
graphite	*R*_int_ = 0.058
ω and φ scans	θ_max_ = 27.5°, θ_min_ = 1.6°
Absorption correction: multi-scan (*SORTAV*; Blessing, 1997)	*h* = −15→15
*T*_min_ = 0.961, *T*_max_ = 0.976	*k* = −32→32
16724 measured reflections	*l* = −33→33

### Refinement

**Table:**

Refinement on *F*^2^	Primary atom site location: structure-invariant direct methods
Least-squares matrix: full	Secondary atom site location: difference Fourier map
*R*[*F*^2^ > 2σ(*F*^2^)] = 0.079	Hydrogen site location: inferred from neighbouring sites
*wR*(*F*^2^) = 0.164	H-atom parameters constrained
*S* = 1.13	*w* = 1/[σ^2^(*F*_o_^2^) + (0.0236*P*)^2^ + 17.5304*P*] where *P* = (*F*_o_^2^ + 2*F*_c_^2^)/3
8869 reflections	(Δ/σ)_max_ < 0.001
543 parameters	Δρ_max_ = 0.53 e Å^−^^3^
0 restraints	Δρ_min_ = −0.45 e Å^−^^3^

### Special details

**Table:**

Geometry. All e.s.d.'s (except the e.s.d. in the dihedral angle between two l.s. planes) are estimated using the full covariance matrix. The cell e.s.d.'s are taken into account individually in the estimation of e.s.d.'s in distances, angles and torsion angles; correlations between e.s.d.'s in cell parameters are only used when they are defined by crystal symmetry. An approximate (isotropic) treatment of cell e.s.d.'s is used for estimating e.s.d.'s involving l.s. planes.
Refinement. Refinement of *F*^2^ against ALL reflections. The weighted *R*-factor *wR* and goodness of fit *S* are based on *F*^2^, conventional *R*-factors *R* are based on *F*, with *F* set to zero for negative *F*^2^. The threshold expression of *F*^2^ > σ(*F*^2^) is used only for calculating *R*-factors(gt) *etc*. and is not relevant to the choice of reflections for refinement. *R*-factors based on *F*^2^ are statistically about twice as large as those based on *F*, and *R*- factors based on ALL data will be even larger.

### Fractional atomic coordinates and isotropic or equivalent isotropic displacement parameters (Å^2^)

**Table:**

	*x*	*y*	*z*	*U*_iso_*/*U*_eq_	
S1	0.66138 (7)	0.09623 (3)	0.72876 (3)	0.0309 (2)	
S2	0.62373 (8)	−0.15753 (4)	0.53600 (3)	0.0381 (2)	
O1	0.5677 (2)	0.11000 (10)	0.75941 (10)	0.0396 (6)	
O2	0.7437 (2)	0.13609 (10)	0.72052 (10)	0.0437 (7)	
O3	0.5218 (2)	−0.05972 (10)	0.71527 (10)	0.0374 (6)	
H3O	0.5299	−0.0770	0.7432	0.045*	
O4	0.5978 (2)	−0.08015 (10)	0.80376 (10)	0.0396 (6)	
O5	0.7262 (2)	−0.14821 (11)	0.51155 (11)	0.0540 (8)	
O6	0.5474 (3)	−0.11417 (10)	0.54076 (10)	0.0497 (7)	
O7	0.7200 (2)	−0.32119 (10)	0.55411 (10)	0.0408 (6)	
H7O	0.7162	−0.3367	0.5250	0.049*	
O8	0.6761 (2)	−0.33403 (10)	0.46026 (10)	0.0425 (6)	
N1	0.7180 (2)	0.04324 (11)	0.75556 (10)	0.0263 (6)	
N2	0.9770 (3)	0.08686 (16)	0.52782 (14)	0.0597 (10)	
N3	0.5641 (2)	−0.20673 (11)	0.50442 (10)	0.0327 (7)	
N4	0.3416 (3)	−0.15139 (16)	0.72641 (14)	0.0588 (10)	
C1	0.6164 (3)	0.07036 (14)	0.66852 (13)	0.0306 (7)	
C2	0.6175 (3)	0.10096 (16)	0.62378 (14)	0.0409 (9)	
H2	0.6445	0.1368	0.6243	0.049*	
C3	0.5782 (4)	0.07843 (19)	0.57778 (15)	0.0507 (11)	
H3	0.5794	0.0989	0.5464	0.061*	
C4	0.5379 (3)	0.02693 (19)	0.57723 (15)	0.0499 (11)	
H4	0.5101	0.0122	0.5457	0.060*	
C5	0.5374 (3)	−0.00393 (16)	0.62239 (14)	0.0378 (9)	
H5	0.5089	−0.0395	0.6217	0.045*	
C6	0.5784 (3)	0.01727 (14)	0.66865 (13)	0.0290 (7)	
C7	0.5841 (3)	−0.01593 (13)	0.71623 (12)	0.0271 (7)	
C8	0.6473 (3)	−0.00275 (12)	0.75820 (12)	0.0263 (7)	
C9	0.6458 (3)	−0.03566 (14)	0.80539 (13)	0.0316 (8)	
C10	0.6942 (3)	−0.01798 (14)	0.85579 (13)	0.0305 (7)	
C11	0.6997 (3)	0.03570 (16)	0.87115 (14)	0.0411 (9)	
H11	0.6787	0.0632	0.8474	0.049*	
C12	0.7352 (4)	0.04952 (18)	0.92032 (15)	0.0491 (10)	
H12	0.7380	0.0864	0.9303	0.059*	
C13	0.7665 (3)	0.0104 (2)	0.95496 (15)	0.0508 (11)	
H13	0.7906	0.0199	0.9890	0.061*	
C14	0.7628 (4)	−0.04306 (19)	0.93996 (16)	0.0564 (12)	
H14	0.7860	−0.0704	0.9635	0.068*	
C15	0.7258 (3)	−0.05697 (17)	0.89121 (15)	0.0452 (10)	
H15	0.7220	−0.0939	0.8817	0.054*	
C16	0.8353 (3)	0.03171 (15)	0.74483 (13)	0.0336 (8)	
H16A	0.8620	0.0055	0.7710	0.040*	
H16B	0.8773	0.0654	0.7493	0.040*	
C17	0.8573 (3)	0.00952 (15)	0.69091 (13)	0.0328 (8)	
C18	0.8915 (3)	0.04363 (15)	0.65099 (14)	0.0345 (8)	
H18	0.9022	0.0809	0.6577	0.041*	
C19	0.9100 (3)	0.02299 (16)	0.60115 (14)	0.0375 (8)	
C20	0.8937 (3)	−0.03093 (16)	0.59040 (15)	0.0422 (9)	
H20	0.9052	−0.0445	0.5561	0.051*	
C21	0.8604 (3)	−0.06509 (17)	0.63033 (17)	0.0480 (10)	
H21	0.8494	−0.1023	0.6235	0.058*	
C22	0.8431 (3)	−0.04491 (16)	0.68026 (15)	0.0393 (9)	
H22	0.8214	−0.0687	0.7074	0.047*	
C23	0.9475 (3)	0.05877 (17)	0.56028 (15)	0.0439 (9)	
C24	0.6470 (3)	−0.18666 (14)	0.59754 (13)	0.0321 (8)	
C25	0.6401 (3)	−0.15611 (16)	0.64282 (14)	0.0398 (9)	
H25	0.6238	−0.1187	0.6411	0.048*	
C26	0.6573 (3)	−0.18092 (18)	0.69031 (15)	0.0466 (10)	
H26	0.6527	−0.1605	0.7216	0.056*	
C27	0.6813 (3)	−0.23523 (18)	0.69267 (15)	0.0490 (11)	
H27	0.6938	−0.2519	0.7255	0.059*	
C28	0.6870 (3)	−0.26550 (16)	0.64761 (14)	0.0398 (9)	
H28	0.7031	−0.3029	0.6497	0.048*	
C29	0.6696 (3)	−0.24188 (15)	0.59913 (13)	0.0328 (8)	
C30	0.6720 (3)	−0.27439 (14)	0.55122 (13)	0.0325 (8)	
C31	0.6264 (3)	−0.25614 (14)	0.50470 (13)	0.0307 (7)	
C32	0.6338 (3)	−0.28706 (15)	0.45824 (14)	0.0339 (8)	
C33	0.5929 (3)	−0.26812 (17)	0.40689 (14)	0.0414 (9)	
C34	0.6057 (4)	−0.21534 (18)	0.39001 (15)	0.0493 (11)	
H34	0.6391	−0.1893	0.4120	0.059*	
C35	0.5691 (4)	−0.2010 (2)	0.34075 (16)	0.0693 (16)	
H35	0.5789	−0.1652	0.3284	0.083*	
C36	0.5181 (5)	−0.2390 (3)	0.30966 (19)	0.095 (2)	
H36	0.4909	−0.2288	0.2764	0.114*	
C37	0.5062 (5)	−0.2912 (3)	0.3262 (2)	0.093 (2)	
H37	0.4717	−0.3170	0.3043	0.112*	
C38	0.5443 (4)	−0.3061 (2)	0.37450 (18)	0.0625 (13)	
H38	0.5375	−0.3425	0.3858	0.075*	
C39	0.4440 (3)	−0.21431 (16)	0.50995 (13)	0.0379 (9)	
H39A	0.4183	−0.2390	0.4822	0.046*	
H39B	0.4075	−0.1791	0.5048	0.046*	
C40	0.4112 (3)	−0.23689 (15)	0.56239 (13)	0.0331 (8)	
C41	0.3888 (3)	−0.20189 (15)	0.60331 (13)	0.0330 (8)	
H41	0.3861	−0.1641	0.5972	0.040*	
C42	0.3703 (3)	−0.22178 (16)	0.65298 (14)	0.0372 (8)	
C43	0.3724 (3)	−0.27700 (17)	0.66260 (16)	0.0483 (10)	
H43	0.3605	−0.2906	0.6968	0.058*	
C44	0.3921 (3)	−0.31156 (17)	0.62160 (17)	0.0502 (10)	
H44	0.3929	−0.3494	0.6276	0.060*	
C45	0.4107 (3)	−0.29198 (16)	0.57163 (16)	0.0415 (9)	
H45	0.4232	−0.3164	0.5437	0.050*	
C46	0.3519 (3)	−0.18360 (17)	0.69450 (15)	0.0434 (9)	

### Atomic displacement parameters (Å^2^)

**Table:**

	*U*^11^	*U*^22^	*U*^33^	*U*^12^	*U*^13^	*U*^23^
S1	0.0379 (5)	0.0250 (4)	0.0298 (4)	−0.0035 (4)	−0.0008 (4)	0.0020 (3)
S2	0.0559 (6)	0.0277 (5)	0.0305 (4)	0.0016 (4)	0.0034 (4)	0.0030 (4)
O1	0.0445 (15)	0.0310 (13)	0.0432 (15)	0.0048 (11)	0.0059 (12)	−0.0061 (11)
O2	0.0533 (16)	0.0319 (14)	0.0459 (15)	−0.0144 (12)	−0.0033 (13)	0.0076 (12)
O3	0.0440 (15)	0.0278 (13)	0.0405 (15)	−0.0091 (11)	0.0007 (12)	−0.0025 (11)
O4	0.0459 (15)	0.0277 (13)	0.0450 (15)	−0.0075 (11)	−0.0002 (12)	0.0096 (11)
O5	0.066 (2)	0.0449 (17)	0.0514 (17)	−0.0127 (15)	0.0158 (15)	0.0044 (14)
O6	0.084 (2)	0.0293 (14)	0.0359 (15)	0.0147 (14)	−0.0030 (14)	−0.0010 (11)
O7	0.0501 (16)	0.0322 (14)	0.0400 (14)	0.0122 (12)	−0.0021 (12)	0.0000 (11)
O8	0.0481 (16)	0.0334 (14)	0.0460 (15)	0.0069 (12)	0.0071 (12)	−0.0063 (12)
N1	0.0259 (14)	0.0266 (14)	0.0264 (14)	−0.0030 (11)	−0.0022 (11)	0.0050 (11)
N2	0.073 (3)	0.062 (2)	0.044 (2)	0.000 (2)	0.0116 (19)	0.0078 (19)
N3	0.0446 (18)	0.0295 (15)	0.0239 (14)	0.0095 (13)	0.0012 (12)	0.0004 (12)
N4	0.075 (3)	0.062 (2)	0.0398 (19)	−0.018 (2)	0.0150 (18)	−0.0064 (18)
C1	0.0307 (18)	0.0320 (18)	0.0293 (17)	0.0013 (15)	−0.0002 (14)	0.0034 (14)
C2	0.042 (2)	0.040 (2)	0.041 (2)	0.0005 (17)	0.0006 (17)	0.0127 (17)
C3	0.054 (3)	0.066 (3)	0.032 (2)	0.003 (2)	−0.0057 (18)	0.015 (2)
C4	0.053 (3)	0.070 (3)	0.0266 (19)	0.002 (2)	−0.0075 (17)	−0.0055 (19)
C5	0.041 (2)	0.041 (2)	0.0321 (19)	−0.0009 (17)	−0.0051 (16)	−0.0060 (16)
C6	0.0267 (17)	0.0306 (18)	0.0297 (17)	0.0068 (14)	−0.0009 (13)	−0.0022 (14)
C7	0.0282 (17)	0.0220 (16)	0.0311 (17)	−0.0005 (13)	0.0042 (13)	−0.0043 (13)
C8	0.0287 (17)	0.0228 (15)	0.0275 (17)	0.0014 (13)	0.0033 (13)	0.0037 (12)
C9	0.0306 (18)	0.0299 (18)	0.0341 (18)	0.0021 (15)	0.0029 (14)	0.0037 (14)
C10	0.0308 (18)	0.0296 (18)	0.0312 (17)	0.0035 (14)	0.0056 (14)	0.0062 (14)
C11	0.054 (2)	0.037 (2)	0.0326 (19)	0.0121 (18)	0.0005 (17)	0.0055 (16)
C12	0.061 (3)	0.051 (3)	0.035 (2)	0.004 (2)	−0.0004 (19)	−0.0057 (19)
C13	0.053 (3)	0.071 (3)	0.0281 (19)	−0.005 (2)	−0.0031 (18)	0.005 (2)
C14	0.067 (3)	0.061 (3)	0.042 (2)	−0.011 (2)	−0.017 (2)	0.025 (2)
C15	0.056 (3)	0.039 (2)	0.041 (2)	−0.0064 (19)	−0.0116 (19)	0.0138 (17)
C16	0.0257 (17)	0.044 (2)	0.0316 (18)	−0.0042 (15)	−0.0015 (14)	0.0038 (16)
C17	0.0225 (17)	0.043 (2)	0.0323 (18)	0.0025 (15)	−0.0010 (14)	0.0029 (15)
C18	0.0290 (18)	0.037 (2)	0.0372 (19)	−0.0005 (15)	0.0028 (15)	−0.0010 (15)
C19	0.0308 (19)	0.046 (2)	0.0358 (19)	0.0019 (16)	0.0050 (15)	0.0011 (17)
C20	0.042 (2)	0.045 (2)	0.040 (2)	0.0006 (18)	0.0017 (17)	−0.0091 (18)
C21	0.048 (2)	0.036 (2)	0.060 (3)	0.0004 (19)	0.003 (2)	−0.0057 (19)
C22	0.032 (2)	0.041 (2)	0.045 (2)	0.0039 (17)	0.0039 (16)	0.0090 (17)
C23	0.045 (2)	0.051 (3)	0.036 (2)	0.0059 (19)	0.0039 (17)	−0.0040 (19)
C24	0.0323 (19)	0.0337 (19)	0.0302 (17)	−0.0019 (15)	−0.0036 (14)	0.0031 (14)
C25	0.042 (2)	0.039 (2)	0.039 (2)	0.0022 (17)	−0.0084 (17)	−0.0082 (16)
C26	0.052 (2)	0.054 (3)	0.033 (2)	0.005 (2)	−0.0125 (18)	−0.0113 (18)
C27	0.051 (2)	0.063 (3)	0.032 (2)	0.010 (2)	−0.0112 (18)	0.0040 (19)
C28	0.044 (2)	0.039 (2)	0.036 (2)	0.0098 (17)	−0.0068 (16)	0.0038 (16)
C29	0.0276 (17)	0.039 (2)	0.0316 (18)	0.0006 (15)	−0.0014 (14)	−0.0010 (15)
C30	0.0318 (18)	0.0308 (18)	0.0348 (18)	0.0009 (15)	0.0022 (15)	0.0024 (15)
C31	0.0357 (18)	0.0287 (18)	0.0276 (16)	0.0036 (15)	0.0017 (14)	0.0002 (13)
C32	0.0304 (18)	0.036 (2)	0.0357 (18)	−0.0008 (15)	0.0042 (15)	−0.0038 (15)
C33	0.040 (2)	0.053 (2)	0.0313 (19)	0.0142 (18)	0.0005 (16)	−0.0107 (17)
C34	0.057 (3)	0.058 (3)	0.034 (2)	0.013 (2)	0.0097 (19)	−0.0008 (19)
C35	0.087 (4)	0.088 (4)	0.032 (2)	0.043 (3)	0.009 (2)	0.010 (2)
C36	0.120 (5)	0.128 (6)	0.037 (3)	0.071 (5)	−0.021 (3)	−0.022 (3)
C37	0.116 (5)	0.098 (5)	0.066 (4)	0.046 (4)	−0.042 (3)	−0.042 (3)
C38	0.071 (3)	0.066 (3)	0.050 (3)	0.020 (3)	−0.017 (2)	−0.023 (2)
C39	0.040 (2)	0.045 (2)	0.0282 (18)	0.0172 (17)	−0.0049 (15)	−0.0058 (16)
C40	0.0270 (17)	0.039 (2)	0.0328 (18)	0.0045 (15)	−0.0042 (14)	−0.0005 (15)
C41	0.0320 (19)	0.0333 (19)	0.0337 (18)	0.0031 (15)	−0.0014 (15)	0.0003 (15)
C42	0.036 (2)	0.043 (2)	0.0326 (18)	−0.0082 (17)	−0.0006 (15)	−0.0003 (16)
C43	0.053 (3)	0.046 (2)	0.045 (2)	−0.013 (2)	−0.001 (2)	0.0074 (19)
C44	0.049 (3)	0.038 (2)	0.063 (3)	−0.0089 (19)	−0.002 (2)	0.006 (2)
C45	0.035 (2)	0.039 (2)	0.050 (2)	−0.0032 (17)	−0.0028 (17)	−0.0092 (18)
C46	0.048 (2)	0.048 (2)	0.035 (2)	−0.0126 (19)	0.0075 (17)	0.0014 (18)

### Geometric parameters (Å, °)

**Table:**

S1—O2	1.430 (3)	C17—C18	1.392 (5)
S1—O1	1.434 (3)	C18—C19	1.394 (5)
S1—N1	1.638 (3)	C18—H18	0.9500
S1—C1	1.760 (3)	C19—C20	1.380 (5)
S2—O5	1.425 (3)	C19—C23	1.447 (5)
S2—O6	1.432 (3)	C20—C21	1.389 (6)
S2—N3	1.637 (3)	C20—H20	0.9500
S2—C24	1.757 (3)	C21—C22	1.389 (5)
O3—C7	1.329 (4)	C21—H21	0.9500
O3—H3O	0.8400	C22—H22	0.9500
O4—C9	1.252 (4)	C24—C25	1.388 (5)
O7—C30	1.304 (4)	C24—C29	1.398 (5)
O7—H7O	0.8400	C25—C26	1.379 (5)
O8—C32	1.276 (4)	C25—H25	0.9500
N1—C8	1.435 (4)	C26—C27	1.380 (6)
N1—C16	1.495 (4)	C26—H26	0.9500
N2—C23	1.143 (5)	C27—C28	1.378 (5)
N3—C31	1.445 (4)	C27—H27	0.9500
N3—C39	1.495 (5)	C28—C29	1.389 (5)
N4—C46	1.150 (5)	C28—H28	0.9500
C1—C2	1.374 (5)	C29—C30	1.468 (5)
C1—C6	1.397 (5)	C30—C31	1.392 (5)
C2—C3	1.390 (5)	C31—C32	1.419 (5)
C2—H2	0.9500	C32—C33	1.484 (5)
C3—C4	1.370 (6)	C33—C34	1.387 (6)
C3—H3	0.9500	C33—C38	1.391 (6)
C4—C5	1.386 (5)	C34—C35	1.386 (6)
C4—H4	0.9500	C34—H34	0.9500
C5—C6	1.391 (5)	C35—C36	1.384 (8)
C5—H5	0.9500	C35—H35	0.9500
C6—C7	1.472 (5)	C36—C37	1.370 (9)
C7—C8	1.366 (4)	C36—H36	0.9500
C8—C9	1.458 (4)	C37—C38	1.374 (7)
C9—C10	1.487 (5)	C37—H37	0.9500
C10—C15	1.381 (5)	C38—H38	0.9500
C10—C11	1.390 (5)	C39—C40	1.510 (5)
C11—C12	1.376 (5)	C39—H39A	0.9900
C11—H11	0.9500	C39—H39B	0.9900
C12—C13	1.371 (6)	C40—C45	1.386 (5)
C12—H12	0.9500	C40—C41	1.388 (5)
C13—C14	1.380 (6)	C41—C42	1.383 (5)
C13—H13	0.9500	C41—H41	0.9500
C14—C15	1.373 (5)	C42—C43	1.391 (5)
C14—H14	0.9500	C42—C46	1.441 (5)
C15—H15	0.9500	C43—C44	1.377 (6)
C16—C17	1.511 (5)	C43—H43	0.9500
C16—H16A	0.9900	C44—C45	1.388 (6)
C16—H16B	0.9900	C44—H44	0.9500
C17—C22	1.388 (5)	C45—H45	0.9500
			
O2—S1—O1	118.94 (16)	C19—C20—C21	119.1 (4)
O2—S1—N1	108.41 (15)	C19—C20—H20	120.4
O1—S1—N1	107.60 (15)	C21—C20—H20	120.4
O2—S1—C1	110.17 (16)	C22—C21—C20	120.2 (4)
O1—S1—C1	108.35 (16)	C22—C21—H21	119.9
N1—S1—C1	102.02 (15)	C20—C21—H21	119.9
O5—S2—O6	119.62 (18)	C17—C22—C21	120.8 (4)
O5—S2—N3	107.42 (17)	C17—C22—H22	119.6
O6—S2—N3	107.96 (17)	C21—C22—H22	119.6
O5—S2—C24	108.49 (18)	N2—C23—C19	179.7 (5)
O6—S2—C24	109.82 (16)	C25—C24—C29	121.5 (3)
N3—S2—C24	102.09 (16)	C25—C24—S2	121.0 (3)
C7—O3—H3O	109.5	C29—C24—S2	117.5 (3)
C30—O7—H7O	109.5	C26—C25—C24	118.9 (4)
C8—N1—C16	116.1 (3)	C26—C25—H25	120.5
C8—N1—S1	113.6 (2)	C24—C25—H25	120.5
C16—N1—S1	119.1 (2)	C25—C26—C27	120.4 (4)
C31—N3—C39	114.6 (3)	C25—C26—H26	119.8
C31—N3—S2	113.1 (2)	C27—C26—H26	119.8
C39—N3—S2	119.3 (2)	C28—C27—C26	120.4 (4)
C2—C1—C6	121.7 (3)	C28—C27—H27	119.8
C2—C1—S1	121.8 (3)	C26—C27—H27	119.8
C6—C1—S1	116.5 (2)	C27—C28—C29	120.7 (4)
C1—C2—C3	118.8 (4)	C27—C28—H28	119.7
C1—C2—H2	120.6	C29—C28—H28	119.7
C3—C2—H2	120.6	C28—C29—C24	118.0 (3)
C4—C3—C2	120.6 (4)	C28—C29—C30	120.8 (3)
C4—C3—H3	119.7	C24—C29—C30	121.2 (3)
C2—C3—H3	119.7	O7—C30—C31	121.3 (3)
C3—C4—C5	120.5 (4)	O7—C30—C29	116.7 (3)
C3—C4—H4	119.7	C31—C30—C29	121.9 (3)
C5—C4—H4	119.7	C30—C31—C32	121.1 (3)
C4—C5—C6	120.0 (4)	C30—C31—N3	119.6 (3)
C4—C5—H5	120.0	C32—C31—N3	119.2 (3)
C6—C5—H5	120.0	O8—C32—C31	119.1 (3)
C5—C6—C1	118.4 (3)	O8—C32—C33	117.6 (3)
C5—C6—C7	120.7 (3)	C31—C32—C33	123.4 (3)
C1—C6—C7	120.9 (3)	C34—C33—C38	120.2 (4)
O3—C7—C8	122.5 (3)	C34—C33—C32	122.4 (4)
O3—C7—C6	114.5 (3)	C38—C33—C32	117.3 (4)
C8—C7—C6	123.0 (3)	C35—C34—C33	119.2 (4)
C7—C8—N1	119.8 (3)	C35—C34—H34	120.4
C7—C8—C9	120.7 (3)	C33—C34—H34	120.4
N1—C8—C9	119.4 (3)	C36—C35—C34	119.8 (5)
O4—C9—C8	118.1 (3)	C36—C35—H35	120.1
O4—C9—C10	118.5 (3)	C34—C35—H35	120.1
C8—C9—C10	123.3 (3)	C37—C36—C35	120.9 (5)
C15—C10—C11	118.1 (3)	C37—C36—H36	119.5
C15—C10—C9	118.4 (3)	C35—C36—H36	119.5
C11—C10—C9	123.2 (3)	C36—C37—C38	119.8 (5)
C12—C11—C10	120.9 (4)	C36—C37—H37	120.1
C12—C11—H11	119.6	C38—C37—H37	120.1
C10—C11—H11	119.6	C37—C38—C33	120.1 (5)
C13—C12—C11	120.3 (4)	C37—C38—H38	120.0
C13—C12—H12	119.8	C33—C38—H38	120.0
C11—C12—H12	119.8	N3—C39—C40	113.3 (3)
C12—C13—C14	119.4 (4)	N3—C39—H39A	108.9
C12—C13—H13	120.3	C40—C39—H39A	108.9
C14—C13—H13	120.3	N3—C39—H39B	108.9
C15—C14—C13	120.4 (4)	C40—C39—H39B	108.9
C15—C14—H14	119.8	H39A—C39—H39B	107.7
C13—C14—H14	119.8	C45—C40—C41	119.1 (3)
C14—C15—C10	121.0 (4)	C45—C40—C39	121.2 (3)
C14—C15—H15	119.5	C41—C40—C39	119.5 (3)
C10—C15—H15	119.5	C42—C41—C40	120.2 (3)
N1—C16—C17	114.2 (3)	C42—C41—H41	119.9
N1—C16—H16A	108.7	C40—C41—H41	119.9
C17—C16—H16A	108.7	C41—C42—C43	120.7 (4)
N1—C16—H16B	108.7	C41—C42—C46	118.0 (3)
C17—C16—H16B	108.7	C43—C42—C46	121.3 (4)
H16A—C16—H16B	107.6	C44—C43—C42	118.7 (4)
C22—C17—C18	119.0 (3)	C44—C43—H43	120.6
C22—C17—C16	120.7 (3)	C42—C43—H43	120.6
C18—C17—C16	120.3 (3)	C43—C44—C45	121.0 (4)
C17—C18—C19	119.9 (4)	C43—C44—H44	119.5
C17—C18—H18	120.0	C45—C44—H44	119.5
C19—C18—H18	120.0	C40—C45—C44	120.2 (4)
C20—C19—C18	121.0 (4)	C40—C45—H45	119.9
C20—C19—C23	119.7 (4)	C44—C45—H45	119.9
C18—C19—C23	119.3 (4)	N4—C46—C42	176.3 (4)
			
O2—S1—N1—C8	−170.7 (2)	C18—C19—C20—C21	−1.3 (6)
O1—S1—N1—C8	59.5 (3)	C23—C19—C20—C21	178.4 (4)
C1—S1—N1—C8	−54.4 (2)	C19—C20—C21—C22	0.4 (6)
O2—S1—N1—C16	−28.2 (3)	C18—C17—C22—C21	−1.4 (5)
O1—S1—N1—C16	−158.1 (2)	C16—C17—C22—C21	178.2 (3)
C1—S1—N1—C16	88.0 (3)	C20—C21—C22—C17	0.9 (6)
O5—S2—N3—C31	59.5 (3)	O5—S2—C24—C25	102.0 (3)
O6—S2—N3—C31	−170.3 (2)	O6—S2—C24—C25	−30.4 (4)
C24—S2—N3—C31	−54.6 (3)	N3—S2—C24—C25	−144.8 (3)
O5—S2—N3—C39	−161.3 (3)	O5—S2—C24—C29	−79.7 (3)
O6—S2—N3—C39	−31.0 (3)	O6—S2—C24—C29	147.9 (3)
C24—S2—N3—C39	84.7 (3)	N3—S2—C24—C29	33.6 (3)
O2—S1—C1—C2	−29.8 (4)	C29—C24—C25—C26	0.7 (6)
O1—S1—C1—C2	101.9 (3)	S2—C24—C25—C26	179.0 (3)
N1—S1—C1—C2	−144.8 (3)	C24—C25—C26—C27	0.2 (6)
O2—S1—C1—C6	151.0 (3)	C25—C26—C27—C28	−0.7 (7)
O1—S1—C1—C6	−77.4 (3)	C26—C27—C28—C29	0.4 (6)
N1—S1—C1—C6	36.0 (3)	C27—C28—C29—C24	0.4 (6)
C6—C1—C2—C3	0.8 (6)	C27—C28—C29—C30	−177.8 (4)
S1—C1—C2—C3	−178.4 (3)	C25—C24—C29—C28	−1.0 (5)
C1—C2—C3—C4	0.9 (6)	S2—C24—C29—C28	−179.3 (3)
C2—C3—C4—C5	−1.1 (7)	C25—C24—C29—C30	177.3 (3)
C3—C4—C5—C6	−0.3 (6)	S2—C24—C29—C30	−1.1 (5)
C4—C5—C6—C1	2.0 (5)	C28—C29—C30—O7	−18.8 (5)
C4—C5—C6—C7	−176.5 (3)	C24—C29—C30—O7	163.0 (3)
C2—C1—C6—C5	−2.2 (5)	C28—C29—C30—C31	161.9 (3)
S1—C1—C6—C5	177.0 (3)	C24—C29—C30—C31	−16.4 (5)
C2—C1—C6—C7	176.3 (3)	O7—C30—C31—C32	−2.2 (5)
S1—C1—C6—C7	−4.5 (4)	C29—C30—C31—C32	177.1 (3)
C5—C6—C7—O3	−17.8 (5)	O7—C30—C31—N3	173.5 (3)
C1—C6—C7—O3	163.8 (3)	C29—C30—C31—N3	−7.2 (5)
C5—C6—C7—C8	162.8 (3)	C39—N3—C31—C30	−95.1 (4)
C1—C6—C7—C8	−15.6 (5)	S2—N3—C31—C30	46.1 (4)
O3—C7—C8—N1	176.1 (3)	C39—N3—C31—C32	80.7 (4)
C6—C7—C8—N1	−4.5 (5)	S2—N3—C31—C32	−138.0 (3)
O3—C7—C8—C9	−2.4 (5)	C30—C31—C32—O8	5.2 (5)
C6—C7—C8—C9	176.9 (3)	N3—C31—C32—O8	−170.6 (3)
C16—N1—C8—C7	−100.3 (3)	C30—C31—C32—C33	−176.0 (3)
S1—N1—C8—C7	43.3 (4)	N3—C31—C32—C33	8.2 (5)
C16—N1—C8—C9	78.3 (4)	O8—C32—C33—C34	−140.8 (4)
S1—N1—C8—C9	−138.1 (3)	C31—C32—C33—C34	40.4 (6)
C7—C8—C9—O4	10.1 (5)	O8—C32—C33—C38	36.6 (5)
N1—C8—C9—O4	−168.4 (3)	C31—C32—C33—C38	−142.2 (4)
C7—C8—C9—C10	−167.1 (3)	C38—C33—C34—C35	0.3 (6)
N1—C8—C9—C10	14.3 (5)	C32—C33—C34—C35	177.6 (4)
O4—C9—C10—C15	26.9 (5)	C33—C34—C35—C36	1.6 (7)
C8—C9—C10—C15	−155.9 (3)	C34—C35—C36—C37	−2.1 (8)
O4—C9—C10—C11	−146.8 (4)	C35—C36—C37—C38	0.6 (10)
C8—C9—C10—C11	30.4 (5)	C36—C37—C38—C33	1.3 (9)
C15—C10—C11—C12	−0.3 (6)	C34—C33—C38—C37	−1.7 (7)
C9—C10—C11—C12	173.4 (4)	C32—C33—C38—C37	−179.1 (4)
C10—C11—C12—C13	0.5 (6)	C31—N3—C39—C40	65.9 (4)
C11—C12—C13—C14	0.4 (7)	S2—N3—C39—C40	−72.8 (4)
C12—C13—C14—C15	−1.4 (7)	N3—C39—C40—C45	−85.6 (4)
C13—C14—C15—C10	1.6 (7)	N3—C39—C40—C41	89.6 (4)
C11—C10—C15—C14	−0.7 (6)	C45—C40—C41—C42	2.4 (5)
C9—C10—C15—C14	−174.7 (4)	C39—C40—C41—C42	−172.9 (3)
C8—N1—C16—C17	67.1 (4)	C40—C41—C42—C43	−0.8 (6)
S1—N1—C16—C17	−74.4 (4)	C40—C41—C42—C46	177.4 (3)
N1—C16—C17—C22	−82.0 (4)	C41—C42—C43—C44	−0.7 (6)
N1—C16—C17—C18	97.5 (4)	C46—C42—C43—C44	−178.9 (4)
C22—C17—C18—C19	0.6 (5)	C42—C43—C44—C45	0.7 (6)
C16—C17—C18—C19	−179.0 (3)	C41—C40—C45—C44	−2.4 (5)
C17—C18—C19—C20	0.8 (5)	C39—C40—C45—C44	172.8 (3)
C17—C18—C19—C23	−178.9 (3)	C43—C44—C45—C40	0.8 (6)

### Hydrogen-bond geometry (Å, °)

**Table:**

*D*—H···*A*	*D*—H	H···*A*	*D*···*A*	*D*—H···*A*
C16—H16A···O3^i^	0.99	2.57	3.382 (4)	139.
C16—H16B···O1^i^	0.99	2.60	3.456 (4)	145.
C28—H28···O2^ii^	0.95	2.45	3.188 (4)	135.
O3—H3O···O4	0.84	1.76	2.503 (4)	146.
O7—H7O···O8	0.84	1.73	2.484 (4)	148.
C11—H11···N1	0.95	2.45	2.974 (4)	115.
C16—H16B···O2	0.99	2.51	2.890 (5)	102.
C34—H34···N3	0.95	2.58	2.982 (5)	106.
C39—H39B···O6	0.99	2.53	2.899 (5)	102.

Symmetry codes: (i) *x*+1/2, *y*, −*z*+3/2; (ii) −*x*+3/2, *y*−1/2, *z*.

## References

[bb1] Ahmad, M., Siddiqui, H. L., Zia-ur-Rehman, M. & Parvez, M. (2010). *Eur. J. Med. Chem.* **45**, 698–704. 10.1016/j.ejmech.2009.11.016 19962218

[bb2] Blessing, R. H. (1997). *J. Appl. Cryst.* **30**, 421–426.

[bb3] Farrugia, L. J. (1997). *J. Appl. Cryst.* **30**, 565.

[bb4] Hooft, R. (1998). *COLLECT* Nonius BV, Delft, The Netherlands.

[bb5] Macrae, C. F., Bruno, I. J., Chisholm, J. A., Edgington, P. R., McCabe, P., Pidcock, E., Rodriguez-Monge, L., Taylor, R., van de Streek, J. & Wood, P. A. (2008). *J. Appl. Cryst.* **41**, 466–470.

[bb6] Otwinowski, Z. & Minor, W. (1997). *Methods in Enzymology*, Vol. 276, *Macromolecular Crystallography*, Part A, edited by C. W. Carter Jr & R. M. Sweet, pp. 307–326. New York: Academic Press.

[bb7] Sheldrick, G. M. (2008). *Acta Cryst.* A**64**, 112–122. 10.1107/S0108767307043930 18156677

[bb8] Siddiqui, W. A., Ahmad, S., Tariq, M. I., Siddiqui, H. L. & Parvez, M. (2008). *Acta Cryst.* C**64**, o4–o6. 10.1107/S0108270107059173 18216455

